# Spatiotemporal dynamic of subtropical forest carbon storage and its resistance and resilience to drought in China

**DOI:** 10.3389/fpls.2023.1067552

**Published:** 2023-01-17

**Authors:** Mengjie Yan, Fangjie Mao, Huaqiang Du, Xuejian Li, Qi Chen, Chi Ni, Zihao Huang, Yanxin Xu, Yulin Gong, Keruo Guo, Jiaqian Sun, Cenheng Xu

**Affiliations:** ^1^ State Key Laboratory of Subtropical Silviculture, Zhejiang Agricultural & Forestry (A & F) University, Hangzhou, China; ^2^ Key Laboratory of Carbon Cycling in Forest Ecosystems and Carbon Sequestration of Zhejiang Province, Zhejiang A & F University, Hangzhou, China; ^3^ School of Environmental and Resources Science, Zhejiang A & F University, Hangzhou, China

**Keywords:** drought, carbon storage, resistance, resilience, subtropical China, forest ecosystem

## Abstract

Subtropical forests are rich in vegetation and have high photosynthetic capacity. China is an important area for the distribution of subtropical forests, evergreen broadleaf forests (EBFs) and evergreen needleleaf forests (ENFs) are two typical vegetation types in subtropical China. Forest carbon storage is an important indicator for measuring the basic characteristics of forest ecosystems and is of great significance for maintaining the global carbon balance. Drought can affect forest activity and may even lead to forest death and the stability characteristics of different forest ecosystems varied after drought events. Therefore, this study used meteorological data to simulate the standardized precipitation evapotranspiration index (SPEI) and the Biome-BGC model to simulate two types of forest carbon storage to quantify the resistance and resilience of EBF and ENF to drought in the subtropical region of China. The results show that: 1) from 1952 to 2019, the interannual drought in subtropical China showed an increasing trend, with five extreme droughts recorded, of which 2011 was the most severe one; 2) the simulated average carbon storage of the EBF and ENF during 1985-2019 were 130.58 t·hm^-2^ and 78.49 t·hm^-2^, respectively. The regions with higher carbon storage of EBF were mainly concentrated in central and southeastern subtropics, where those of ENF mainly distributed in the western subtropic; 3) The median of resistance of EBF was three times higher than that of ENF, indicating the EBF have stronger resistance to extreme drought than ENF. Moreover, the resilience of two typical forest to 2011 extreme drought and the continuous drought events during 2009 - 2011 were similar. The results provided a scientific basis for the response of subtropical forests to drought, and indicating that improve stand quality or expand the plantation of EBF may enhance the resistance to drought in subtropical China, which provided certain reference for forest protection and management under the increasing frequency of drought events in the future.

## 1 Introduction

Carbon storage generally refers to the storage of carbon elements in each carbon pool of the forest ecosystem at a certain point in time, and is the result of years of accumulation in the forest ecosystem (Sun and Liu, 2020). It is not only an important indicator that reflects the basic characteristics of the forest ecological environment (Fang et al., 2014) but also a theoretical basis for evaluating forest structure, function, and production potential (Bonan, 2008; Mitchard, 2018). However, frequent drought induced by climate change greatly affects the carbon sequestration process of forest ecosystem (Schwalm et al., 2012).

At present, the physical process underlying drought impacts on forests has been well studied. In general, drought slows down forest activities and affects forest stability by reducing forest productivity. Extreme drought directly or indirectly affects forest GPP (Xu et al., 2019) and terrestrial carbon sinks (Jung et al., 2017), and may even lead to forest death (Park Williams et al., 2013; Hartmann et al., 2018). However, the response of terrestrial ecosystems to drought is one of the largest uncertainties in the carbon cycle (Reichstein et al., 2013) and is not well represented in current climate-vegetation models (Anderegg et al., 2015). Therefore, analyzing how forests respond and adapt to drought has become a focal issue in the study of extreme events in the context of climate change. Drought monitoring at the spatial scale is generally evaluated by drought indices (Vicente-Serrano et al., 2010; Gobena and Gan, 2013; Center et al., 2017). Among them, the Standardized Precipitation Evapotranspiration Index (SPEI) characterizes the degree of deviation of a region’s dry and wet conditions from the normal year by standardizing the cumulative probability value of the difference between potential evapotranspiration (PET) and precipitation (Xia et al., 2019), and has been widely used in drought detection research around the world (Vicente-Serrano and Trigo, 2011; Zhuang et al., 2013; Lei et al., 2015; Xia et al., 2019).

The effects of drought on ecosystem stability can be expressed using ecosystem resistance and resilience (Tilman and Downing, 1994; De Keersmaecker et al., 2014), which are two factors that fully consider the immediate and legacy effects of drought on forest ecosystems (Ivits et al., 2016; Pennekamp et al., 2018). Resistance expressed as the ability of the ecosystem to maintain its original state under disturbance (Lloret et al., 2007; Van Ruijven and Berendse, 2010), and resilience represents the ability of the ecosystem to recover to a normal state from the disturbance (Holling, 1973). Scholars have previously analyzed the resistance and resilience of species at the biome level based on the perspective of experiments and modeling (Hoover et al., 2014; De Keersmaecker et al., 2015; Gazol et al., 2018; Li et al., 2018). Isbell et al. (2015) defined a dimensionless measure of resistance and resilience of grassland ecosystems by measuring the productivity of grassland systems in North America. Huang and Xia (2019) directly quantified the resistance and resilience of global ecosystems to drought by measuring the ecosystem function change. However, few studies have quantified forest resistance and resilience to drought by measuring changes in forest carbon pool function during and after drought, and the response mechanism of forest carbon pool change to drought has not been clarified.

Forest carbon storage estimation methods commonly include plot survey, remote sensing inversion, and ecosystem model. The sample plot survey seemed as the most accurate method to measure the carbon storage of forest ecosystems, while considerable amount of time costs, manpower, material resources limited restricted its use (Mickler et al., 2002; Chave et al., 2003). The remote sensing inversion could obtain vegetation carbon storage in real time and on a large scale. However, due to technical limitations and the lack of description of plant physiology, the estimation of underground carbon storage still remains large uncertainty (Sun and Liu, 2020). Ecosystem models can mechanistically describe key carbon fixation processes, which is suitable for large-scale research. At present, a large number of carbon storage estimation models have been developed. Among them, the Biome-BGC model is a typical ecosystem process model that can simulate physiological and ecological processes, such as photosynthesis, respiration, and decomposition of ecosystems at different scales, and it is widely used worldwide (Running, 1993; White et al., 2000; Wu et al., 2014).

The subtropical forest ecosystems in the East Asian monsoon region have a net ecosystem productivity of 0.72 Pg C·a^-1^, indicating them play a non-negligible role for mitigating global warming (Yu et al., 2014). China is an important distribution area of subtropical forests in East Asia (Zhou et al., 2003; Yan et al., 2006), in which evergreen broadleaf forest (EBF) and evergreen needleleaf forest (ENF) are two most widely distributed forest types with great carbon sink potential (He et al., 2017; Mao et al., 2022). However, drought happened recent years significantly affects the carbon balance of EBF and ENF ecosystems, such as the extreme drought in 2003 caused a 55% annual NEP decline in the planted ENF of QianYanZhou (Gu et al., 2008), and Song et al. (2017) found the water use efficiency (WUE) greatly increased in the driest year (2009) due to a larger decline in evapotranspiration than gross primary productivity. In addition, due to the complex climatic conditions (Hanson and Weltzin, 2000; Xie et al., 2015), different in drought intensities and duration (Niu et al., 2014; Vicente-Serrano et al., 2014), and various in vegetation physiological (Liu et al., 2013; Li et al., 2020), the impacts of drought on EBF and ENF in subtropical China may have spatial heterogeneous.

This study takes EBF and ENF as the research object, calculated the SPEI in subtropical China, and analyzed the spatiotemporal characteristics of subtropical extreme drought from 1952 to 2019. Then, the driven datasets of Biome-BGC model were collected, and the spatiotemporal evolution trend of vegetation carbon storage of EBF and ENF from 1985 to 2019 were simulated. Finally, extreme droughts and continuous droughts were extracted, and the resistance and resilience of subtropical forests to extreme droughts were analyzed based on the simulated carbon storage. The findings of this study can provide a scientific basis for enhancing the conservation and management of subtropical forests in the context of future global warming, and it has certain enlightenment significance for the forest to resist extreme drought.

## 2 Materials and methods

### 2.1 Study area

The study area is the entire subtropical region of China, which is located to the south of the Qinling Mountains and Huaihe River, north of Leizhou Peninsula, and east of the Hengduan Mountains (22° -34°N, 98° -122°E). The terrain is low in the west and high in the east. China’s subtropics belong to the east coast humid monsoon area, and the region is the warmest and hottest compared to the same latitude, except for desert areas. Moreover, the rainfall in the study region is far more abundant than that in the same latitude worldwide (Yang et al., 2006). The average annual temperature ranges from -1 to 24°C, and the average annual precipitation ranges from 450 to 2125mm. China has preserved the best subtropical evergreen forest ecosystem which is the main component of China’s subtropical forests (Lin et al., 2020) and accounts for approximately 25% of China’s land area. Dominant families in such ecosystems are the *Cyclobalanopsis Oerst*, *Castanopsis Spach*, and *Lithocarpus* of *Fagaceae*.

### 2.2 Data acquisition and processing

#### 2.2.1 Meteorological data

**Figure 1:**
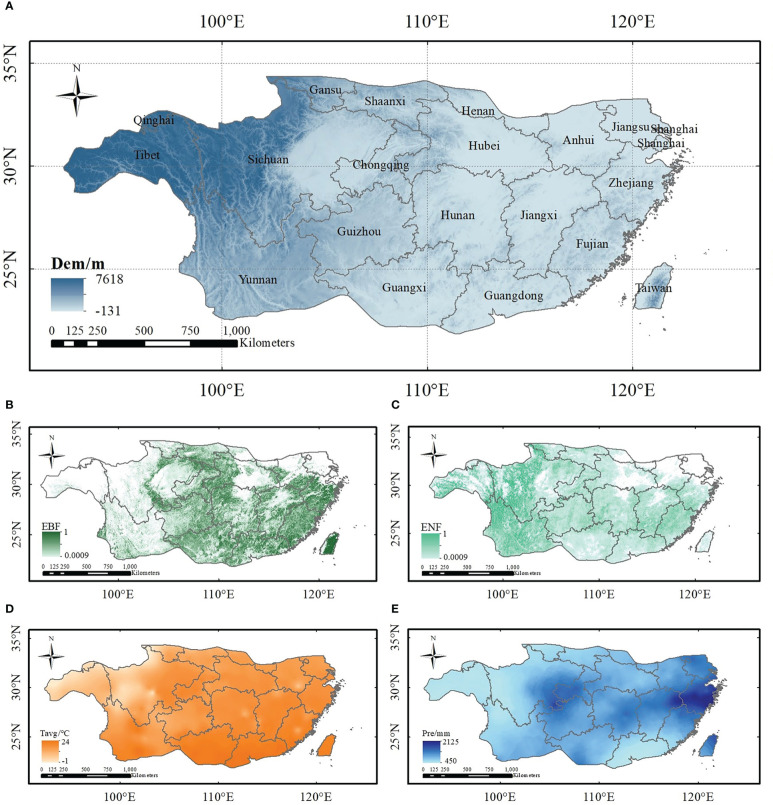
Extent of Subtropical China and related datasets used in this study **(A)** elevation; **(B)** abundance of EBF; **(C)** ENF abundance of ENF; **(D)** annual average temperature; and **(E)** average precipitation).

Meteorological data for the study area from 1952 to 2019, including the daily maximum temperature, minimum temperature, solar radiation, precipitation, relative humidity, and average wind speed, were obtained from the National Meteorological Information Center of the China Meteorological Administration (http://data.cma.cn). The processing steps for the meteorological data were as follows: 1. a spatial resolution of 1 km was used to interpolate meteorological data from 824 meteorological stations using the inverse distance weight method; 2. temperature was corrected based on altitude, assuming a temperature drop rate of 6.5°C·km^-1^ (Cao et al., 2017); Then, solar radiation was simulated using the method of Ju et al. (2006) according to the sunshine duration of each station. 3. meteorological data were obtained for the subtropical region in China through extraction by mask. The average monthly data can be obtained by averaging and summing the corresponding daily scale data (as shown in **Figures 1D, E**).

#### 2.2.2 Elevation data

The elevation data (as shown in **Figure 1A**) used here are from the ASTER Global Digital Elevation Model version 3 (Fujisada et al., 2005), and the spatial resolution of the data is 1°, thus, re-projection of the data must be performed before use. The geographic coordinates of the data were converted to projection coordinates, and the data were resampled to 1 km. Finally, elevation data of the subtropical region of China were obtained by mask clipping.

#### 2.2.3 Soil data

**Figure 2:**
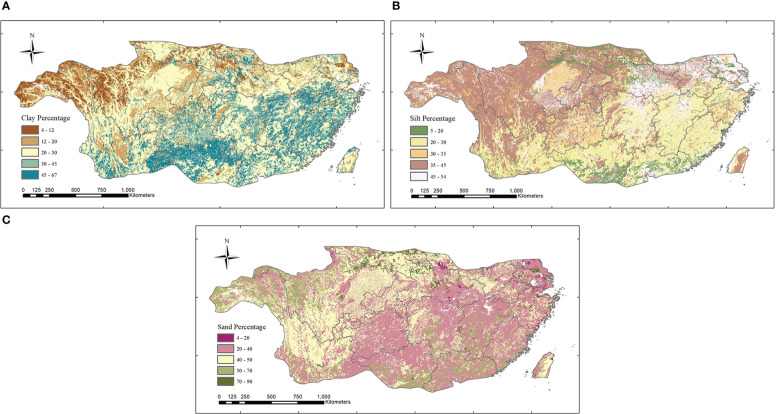
The percentage of clay **(A)**, silt **(B)**, and sand **(C)** of subtropical China.

Subtropical soil data were obtained by Harmonized World Soil Database ver. 1.2 (http://iiasa.acat/Research/LUC/luc07/External-World-soil-database) (Wieder et al., 2014; Xianyong and Hao, 2018), which has a spatial resolution of 0.05^°^. The original data needed to be reprojected and resampled to a resolution of 1 km, and then the soil texture data in the subtropical region of China could be obtained by mask cutting. Finally, data on the clay, sand, and silt particles were extracted according to field values to obtain the percentage of data subtropical clay, sand, and silt in the study area (**Figure 2**).

#### 2.2.4 Subtropical forest abundance data

In this study, the abundance data of subtropical EBF and ENF were used to simulate carbon storage, and these data were obtained from the Global 30 m Fine Land Cover Dynamic Monitoring Product from 1985 to 2020 (Zhang et al., 2021), which were released by the Space Information Innovation Institute of the Chinese Academy of Sciences. This dataset uses full-time series Landsat satellite data and relies on the Google Earth Engine cloud computing platform to achieve a global annual land cover map containing more than 30 land cover types. Before simulating carbon storage, the data were first trimmed to the subtropical region of China, and then the data for the two forest types were extracted according to the fields values. Finally, the forest distribution data at 30 m resolution were linear upscaled to achieve the 1 km fraction data using the local average method (Shang et al., 2013) (**Figures 1B, C**). Because the time interval for land-use type data was five years, the forest abundance in years that were unclassified at the time of simulation was replaced with stand data from neighboring years.

### 2.3 Spatial and temporal patterns and analysis of subtropical drought based on the SPEI

In this study, the SPEI were calculated based on soil heat flux, meteorological data, saturated water vapor pressure, sunshine duration, wind speed, and latitude. The calculation process of the SPEI based on this algorithm is as follows (Zotarelli et al., 2010).

1). The core of the SPEI algorithm is the calculation for PET. The Penman-Monteith (P-M) formula considered heat and aerodynamics factors, and is more consistent with the measured evapotranspiration (Penman, 1948; Jensen et al., 1990). The P-M formula is as follows:


(1) $$PET=\frac{0.408(R_{s}-G)+\gamma \frac{900}{T+273}u_{2}(e_{s}-e_{a})}{\Delta +\gamma (1+0.34u_{2})}$$

where R_s_ is the daily average net radiation flux of the plant surface (MJ·m^-2^·day^-1^); G is the soil heat flux (MJ·m^-2^·day^-1^); γ is the humidity constant (kPa·°C^-1^); T is the average temperature(°C); U_2_ is the average wind speed at 2 m per day (m·day^-1^); and e_s_, e_a_ and Δ are the saturated water vapor pressure (kPa), actual water vapor pressure (kPa) and slope of the saturated water vapor pressure-temperature curve, respectively. R_s_, G, γ, and other parameters were calculated using the FAO-56 P-M updating equation recommended by the FAO (Allen et al., 1998).

2). Calculate the measurement of deficit water balance (D_i_):


(2) $$D_{i}=P_{i}-PET_{i}$$

3). Create a series of cumulative water deficits at different time scales ${\text{D}}_{\text{n}}^{\text{k}}$ :


(3) $$D_{n}^{k}=\sum_{i=0}^{k-1}\left(P_{n-1}-PET_{n-1}\right)\;n\geq k$$

where k is the time scale (generally on month) and n is the number of calculations.

4). Choose the log-logistic probability distribution (negative values can be interpreted and modeled with different shapes for the frequencies of the D series at different timescales) to normalize the D series:


(4) $$F(x)={\left[1+{\left(\frac{\alpha }{x-\gamma }\right)}^{\beta }\right]}^{-1}$$

In formula (4) the following parameters are defined:


(5) $$\alpha =\frac{(w_{0}-2w_{1})\beta }{\Gamma (1+1/\beta )\Gamma (1-1/\beta )}$$

(6) $$\beta =\frac{2w_{1}-w_{0}}{6w_{1}-w_{0}-6w_{2}}$$

(7) $$\gamma =w_{0}-\alpha \Gamma (1+1/\beta )\Gamma (1-1/\beta )$$

where *Γ* is a factorial function and w_0_, w_1_, and w_2_ are the probability-weighted moments of D_i_.


(8) $$w_{s}=\frac{1}{N}\sum_{i=1}^{N}{\left(1-F_{i}\right)}^{s}D_{i}$$

(9) $$F_{i}=\frac{i-0.35}{N}$$

where N is the number of months involved in the calculation.

5). Standardize the cumulative probability density to obtain the SPEI (the standard value of SPEI is 0, and the standard deviation is 1):

First, calculate the size of P:


(10) P=1-F(x)

①

$When\;P\leq 0.5,w=\sqrt{-2ln(P)}$ ,


(11) $$SPEI=w-\frac{c_{0}+c_{1}w+c_{2}w^{2}}{1+d_{1}w+d_{2}w^{2}+d_{3}w^{3}}$$

②

$WhenP>0.5,P=1-P,w=\sqrt{-2ln(P)}$ ,


(12) $$SPEI=-\left(w-\frac{c_{0}+c_{1}w+c_{2}w^{2}}{1+d_{1}w+d_{2}w^{2}+d_{3}w^{3}}\right)$$

In the above equations, c_0 =_ 2.515517, c_1 =_ 0.802853, c_2 =_ 0.010328, d_1 =_ 1.432788, d_2 =_ 0.189269, and d_3 =_ 0.001308.

The SPEI usually has a variety of time scales, such as monthly, seasonal, and annual. Compared to the SPEI with shorter time scales for targeting meteorological and agricultural drought, the SPEI with a longer time scale is more sensitive to hydrological drought (Ivits et al., 2014), moreover, vegetation germination in biological communities is mainly affected by accumulated precipitation in the previous 12 months (Vicente-Serrano, 2006). Therefore, in this study, the SPEI value at the 12-month scale (hereafter referred to as SPEI_12_) was used to characterize the interannual drought in subtropical regions, and the linear regression equation was used to evaluate the trend and characteristics of SPEI_12_ over time based on the following formula:


(13) $$slope=\frac{y\times \sum_{i=1}^{y}i\times SNP_{i}-\sum_{i=1}^{y}i\sum_{i=1}^{y}SNP_{i}}{y\times \sum_{i=1}^{y}i^{2}-{\left(\sum_{i=1}^{y}i\right)}^{2}}$$

where *slope* is the changing trend (when *slope >*0, it indicates that SPEI_12_ is increasing; when *slope*<0, then the SPEI_12_ is decreasing), y is the number of drought years, *i* is the number of years (i=1, 2… n), and SNP_i_ is the SPEI_12_ value of the i_th_ year.

On the spatial scale, the drought classification criteria (**Table 1**) (Center et al., 2017) were used to define the drought conditions among different regions in subtropical China. For the time series, the overall mean value could not meet the criteria for drought classification due to the spatial variation of SPEI_12_. Therefore, in this study, the threshold value of the SPEI_12_ percentile was used to define the annual drought conditions with reference to the precipitation percentile threshold method (Zhai and Pan, 2003), as shown in **Table 2**.

**Table 1:** Drought grade of standardized precipitation evapotranspiration index.

SPEI	Drought levels
-0.5<SPEI	No Drought
-1.0<SPEI≤-0.5	Mild Drought
-0.5<SPEI≤-1	Moderate Drought
-1<SPEI≤-1.5	Severe Drought
-1.5<SPEI≤-2	Extreme Drought

**Table 2:** SPEI percentile threshold for determining water balance conditions.

SPEI Percentile	Conditions
≥90%	Extreme Wet
75%-90%	Moderate wet
25%-75%	Normal
10%-25%	Moderate Drought
≤10%	Extreme Drought

### 2.4 Spatiotemporal simulation and trend analysis of subtropical forest carbon storage

In this study, the carbon storage of vegetation in two types of forests in subtropical China from 1985 to 2019 was simulated with a spatial resolution of 1km and temporal resolution on a daily time steps.

The Biome-BGC model simulates the physiological and ecological processes of vegetation that control the material cycle and energy flow of the ecosystem, including canopy radiation, photosynthesis, stomatal conductance, autotrophic respiration, heterotrophic respiration, phenological dynamics, and evapotranspiration. The model can simulate the energy and carbon-nitrogen water cycles between the atmosphere, vegetation, and soil of the terrestrial ecosystem in daily steps to estimate the storage and flux fluxes among carbon, nitrogen, and water pools (Running, 1993).

The input data to the Biome-BGC model included vegetation abundance data, topographic data, soil data, and meteorological data, which has been described in Section 2.2.

This study used 67 physiological and ecological parameters to run the model. At present, few studies have focused on the physiology and ecology parameters of vegetation. In this study, the proportion of nitrogen in the Rubisco enzyme, litter coefficient at the leaf replacement period, and the carbon-nitrogen distribution ratio of each part were obtained by an iterative method (Lu et al., 2016). In addition, the default ENF or EBF parameters provided by White (White et al., 2000) were adopted if the parameters could not be determined through the literature. Some parameter values are shown in **Table 3**.

**Table 3:** Main parameters input to the Biome-BGC model.

Parameter	EBF	ENF	Unit
Froot turnover	0.7	0.7 (Lu et al., 2016)	a^-1^
Specific leaf area	10	12 (White et al., 2000)	m·Kg C^-1^
The proportion of unstable substances in fine roots	34	34 (Liu and Fei, 2013)	%
Fine root cellulose ratio	44	44 (Liu and Fei, 2013)	%
Fine root lignin ratio	24	22 (Holling et al., 1995)	%
The ratio of nitrogen to Rubisco enzyme	0.08	0.07 (Lu et al., 2016)	kg N Rub·kg N leaf^-1^
Leaf carbon and nitrogen ratio	42	42 (White et al., 2000)	kg C·kg N^-1^
Litter carbon-nitrogen ratio	49	93 (White et al., 2000)	kg C·kg N^-1^
Fine roots carbon-nitrogen ratio	58	58 (White et al., 2000)	kg C·kg N^-1^
Living wood carbon-nitrogen ratio	50	58 (White et al., 2000)	kg C·kg N^-1^
Deadwood carbon-nitrogen ratio	550	730 (White et al., 2000)	kg C·kg N^-1^
The biggest stomatal conductance	0.006	0.006 (White et al., 2000)	m·s^-1^
The surface conductance	0.00006	0.00006 (White et al., 2000)	m·s^-1^
Boundary layer conductance	0.09	0.01 (White et al., 2000)	m·s^-1^

First, the model was spun-up, meaning that the carbon, nitrogen, and water storage of the ecosystem when the annual change in the soil carbon pool was less than 0.0005 kg C·m^-2^ (Thornton, 2010) was used as the initial condition of the simulation, and then the vegetation carbon storage of the EBF and ENF in subtropical China from 1985 to 2019 was simulated.

Based on the simulation results, the linear regression analysis was used to calculate the trend of simulated carbon storage. The equation is similar to Eq. 13, where *slope* is the changing trend (when *slope >*0, carbon storage is increasing, and when *slope*<0, carbon storage is decreasing), y is the number of simulated years, i is the number of years (*i*=1, 2,…, n), and SNP_i_ is the value of carbon storage in the *i*_th_ year.

### 2.5 Evaluation of forest resistance and resilience

Forest ecosystem resistance (Rt) represents the ability of a forest ecosystem to maintain its original state under drought disturbance, whereas forest ecosystem resilience (Rs) describes the ability of a forest to recover from drought disturbance to a normal state (Van Ruijven and Berendse, 2010; De Keersmaecker et al., 2014). These parameters represent functions of the stability of an ecosystem (De Keersmaecker et al., 2014). In this study, formulas 14 and 15 were used to quantitatively explain the Rt and Rs of subtropical forests to drought, where the values of Rt and Rs are unitless to facilitate the comparison of the stability of forest ecosystems with two different levels of productivity.


(14) $$Rt=\frac{\bar{Y_{n}}}{\left|Y_{e}-\bar{Y_{n}}\right|}$$

(15) $$Rs=\left|\frac{Y_{e}-\bar{Y_{n}}}{Y_{e+i}-\bar{Y_{n}}}\right|$$

where Y*_n_* is the carbon storage in the normal year from 1985 to 2019, Y*_e_* is the carbon storage in the extreme drought year, and Y*_e_*_+_ *_i_* is the forest carbon storage in year *i* after the event. Since the effects of drought on forest growth may last for several years and lead to legacy effects (Anderegg et al., 2015; Schwalm et al., 2017), *i* =1, 2, and 4 was used in this study to quantitatively analyze the resilience of carbon storage in the first, second, and fourth years after drought (in addition, the water balance of the forest in the four years after drought should also be considered). A higher Rt value indicates that the forest is more resistant to drought and a higher Rs value means stronger forest resilience (Huang and Xia, 2019).

In this study, we first analyzed the spatiotemporal distribution trend of the SPEI_12_ in the subtropical region of China from 1952 to 2019. Extreme drought years were determined based on the drought time series. Combined with the simulation results of carbon storage in subtropical forests, the resistance and resilience of forests affected by extreme drought were analyzed. As the frequency of drought changes, the acclimation of forests to drought may also change accordingly (Anderegg et al., 2015; Isbell et al., 2015; Anderegg et al., 2020). Therefore, whether continuous drought occurred before and after the drought year was determined in this study, and whether continuous drought affects forest resistance and resilience is also a significant step in this research and discussion.

## 3 Result

### 3.1 Spatiotemporal characteristics of SPEI and drought trend

**Figure 3:**
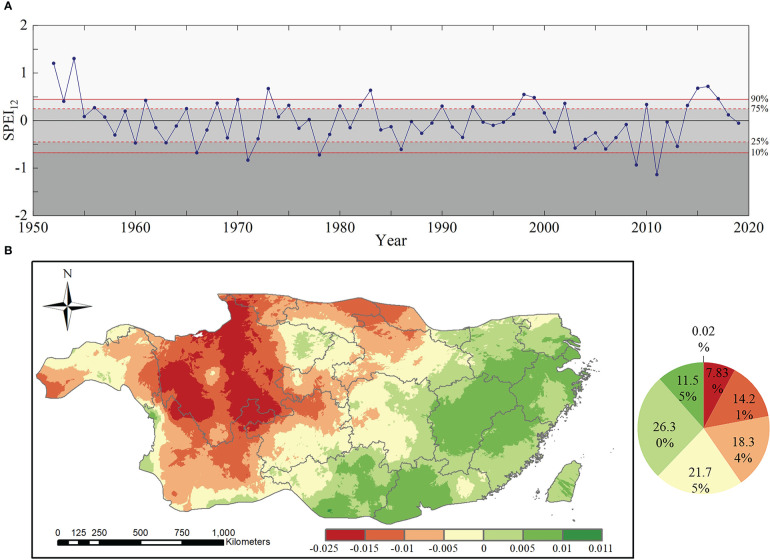
Spatiotemporal trend of SPEI_12_: **(A)** Time series and percentiles of drought conditions of SPEI_12_ from 1952 to 2019 (the solid red lines at the 10th and 90th percentiles represent thresholds for extreme drought and extreme wetness, respectively, and the dashed red lines at the 25th and 75th percentiles represent thresholds for moderate drought and moderate wetness, respectively and different regions represent different drought conditions); and **(B)** spatial variation trend of subtropical drought from 1952 to 2019.

The time series of SPEI_12_ in the subtropical region from 1952 to 2019 are shown in **Figure 3A**. During the 68 years, subtropical forests in China experienced five extreme drought events, which occurred every 13.6 years. According to the spatial variation trends in drought (**Figure 3B**), SPEI_12_ showed a decreasing trend in 62.13% of the region, with an overall decrease of 0.036(10 a)^-1^. **Figure 3B** showed the trends and characteristics of SPEI_12_ with time using the linear regression equation (Eq. 13). The regions with a serious downward trend were mainly distributed in most of the western subtropical region, especially in parts of the Sichuan and Yunnan provinces, with a downward trend of more than 0.15 (10a)^-1^.

According to **Figure 3A**, two extreme drought events occurred after 1985 in 2009 and 2011. Because the SPEI_12_ value was lower in 2011, 2011 was defined as the extreme drought year in this study. Therefore, this study focused on the resistance of subtropical forests in 2011 to drought and their resilience at the first to fourth year after the 2011 extreme drought, which covers the period from 2011 to 2015.

**Figure 4:**
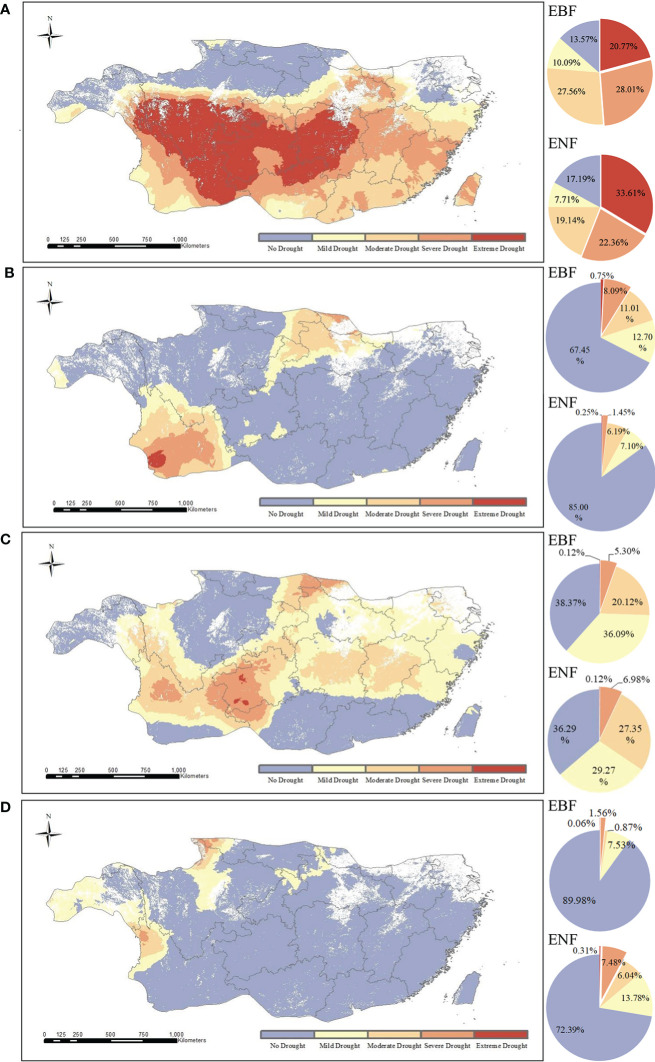
Spatial distribution of different drought levels of two typical subtropical forests in 2011 **(A)**, 2012 **(B)**, 2013 **(C)**, and 2015 **(D)**.

To compare the different drought conditions of the two forest types, we calculated the pixel proportions of the two forest types under different drought conditions (**Figure 4**). According in **Figure 4A**, the areas of extreme and severe drought were concentrated in the central to southwestern region of subtropical China, where the forest cover rate was high. The results for EBF showed that 19.3% suffered from extreme drought (SPEI_12_≤-2) and 26.6% suffered from severe drought (-2< SPEI_12_≤-1.5), while those for ENF showed that 31.9% suffered from extreme drought (SPEI_12_≤-2) and 21.2% suffered from severe drought condition (-2< SPEI_12_≤-1.5). The results showed that most EBF and ENF in subtropical regions of China suffered from severe to extreme drought in 2011.

The value of SPEI12 in 2012 is -0.028, and the water balance condition is normal as shown in **Figure 3**. As shown in **Figure 4B**, only 8.84% of EBF pixels and 1.7% of ENF pixels are under severe to extreme drought conditions in 2012, and the majority of them are under normal water balance condition.

The SPEI_12_ value in 2013 was -0.526, which represents a moderate drought according to the time series. However, according to the spatial distribution (**Figure 4C**), only a few forest pixels in the subtropical region of China were affected by extreme to severe drought in 2013, and the subtropical forests as a whole had normal moisture status.

The SPEI_12_ value was 0.587 in 2015, indicating the absence of drought. Further analysis showed that the vast majority of forest pixels in the EBF (95.34%) and ENF (79.56%) were in the normal to wet state without drought in 2015 (**Figure 4D**).

### 3.2 Spatial and temporal variation trends in carbon storage in subtropical forests

**Figure 5:**
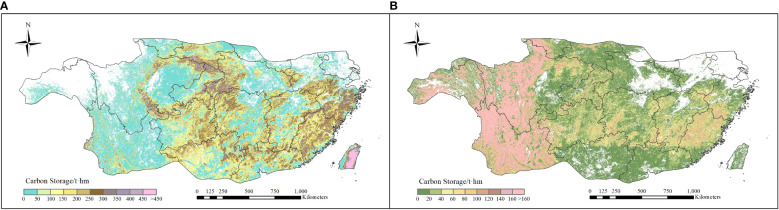
Spatial distribution of average carbon storage for two types of forests during 1985-2019 **(A)** EBF and **(B)** ENF).

The spatial distribution of average vegetation carbon storage of EBF and ENF during 1985-2019 were shown in **Figure 5**. As shown in the figure, the average vegetation carbon storage of EBF and ENF ranged in 124.03 - 143.45 t hm^-2^ (130.58 ± 10.02 t hm^-2^) and 77.21 - 82.59 t hm^-2^ (78.49 ± 8.49 t hm^-2^), respectively. In terms of spatial distribution, the regions with higher carbon storage of EBF were mainly concentrated in central and southeastern subtropics, where those of ENF mainly distributed in the western subtropic, such as Tibet, Yunnan and Sichuan province.

**Figure 6:**
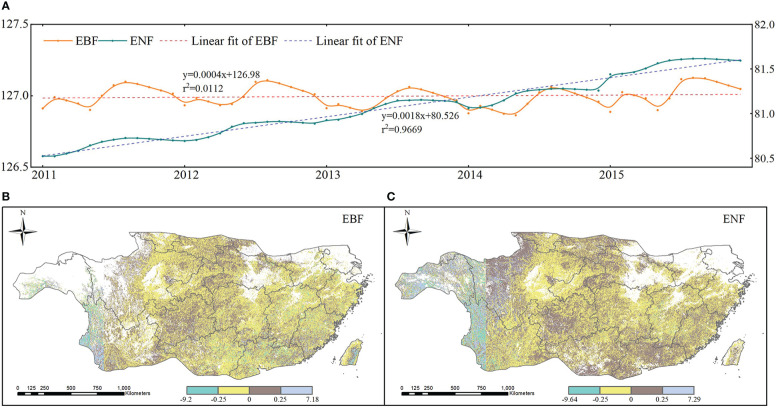
Time serials and spatial trends of carbon storage from 2011 to 2015 **(A)**. Monthly carbon storage; **(B)** trends of EBF; and **(C)**. trends of ENF).

According to the analysis of spatiotemporal characteristics of subtropical drought, follows we mainly focus on the vegetation carbon storage of two types of forest during 2011-2015 for better understanding the resistance and resilience of subtropical forests to drought. The monthly time series and spatial trends of carbon storage of two subtropical forest are shown in **Figure 6**. As shown in **Figure 6A**, the carbon storage of EBF fluctuates within a certain range, whereas the ENF fluctuates relatively little and continues to increase each year. From 2011 to 2015, the mean values of the overall annual trend of carbon storage for the two forest species in subtropical China were -0.042 (EBF) and -0.013 (ENF). The overall carbon storage of subtropical evergreen forests showed a slightly decreasing trend, and the annual variation ranged mainly between -0.25 and 0.25. In subtropical China, 60.5% of the EBF and 51.18% of the ENF pixels showed a downward trend from 2011 to 2015. **Figures 6B, C** shows the trends and characteristics of carbon storage changes over time evaluated using the linear regression equation (Eq. 13). The spatial distribution of the changing trend in carbon storage in the two forests (**Figures 6B, C**) was relatively complex: the two forests showed an overall downward trend; the pixels in the middle and southeast of the EBF showed an overall upward trend, and the pixels from northwest to southeast of the ENF showed an upward trend.

### 3.3 Stability of the EBF and ENF in subtropical China during drought in 2011

To evaluate the stability of forests in subtropical China to drought, we first explored the resistance of the two forest types to drought in 2011 and the resilience of the forests at 1, 2, and 4 years after 2011.

#### 3.3.1 Comparison of resistance of two forest types to drought in 2011

**Figure 7:**
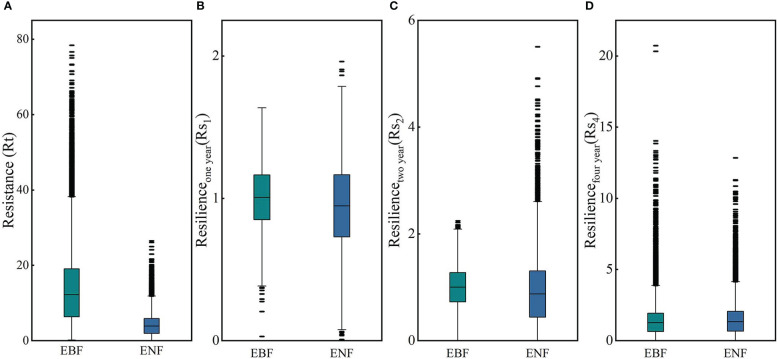
Resistance of the EBF and ENF to drought in 2011 **(A)**, and their resilience to the 2011 extreme drought after the first **(B)**, second **(C)**, and fourth **(D)** year.

**Figure 8:**
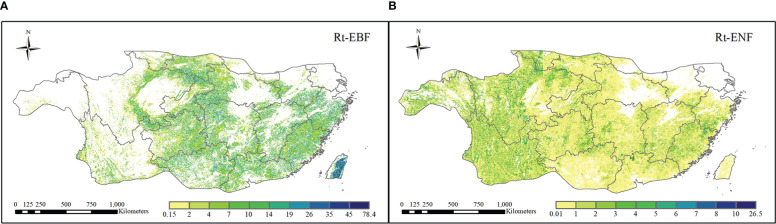
Spatial distribution of resistance to 2011 drought **(A)**. EBF and **(B)** ENF).

The resistance levels of the EBF pixels and ENF pixels for 2011 are shown in **Figure 7A**. According to the calculation results, the ranges of Rt_EBF_ and Rt_ENF_ were different to some extent and had values of 0.15 - 78.40 and 0.01 - 26.47, respectively. The median resistance value of the EBF calculated based on simulated carbon storage was 12.21, and that of the ENF was 3.85. These results clearly show that the resistance of the EBF to the 2011 drought, which was based on carbon storage, was significantly higher than that of the ENF. The spatial distributions of the Rt_EBF_ and Rt_ENF_ are shown in **Figures 8A, B**. In the western region of the subtropical zone, the EBF exhibits weak resistance to drought, while in the central to eastern region, the EBF exhibits strong resistance to drought, gradually increasing from west to east. The overall spatial distribution of ENF resistance was relatively average, with the western subtropical region being slightly stronger than the central and eastern regions. There are some differences in the spatial distributions of the EBF and ENF resistance. The results showed that the two forest types responded differently to the 2011 drought.

#### 3.3.2 Comparison of EBF and ENF resilience

**Figure 9:**
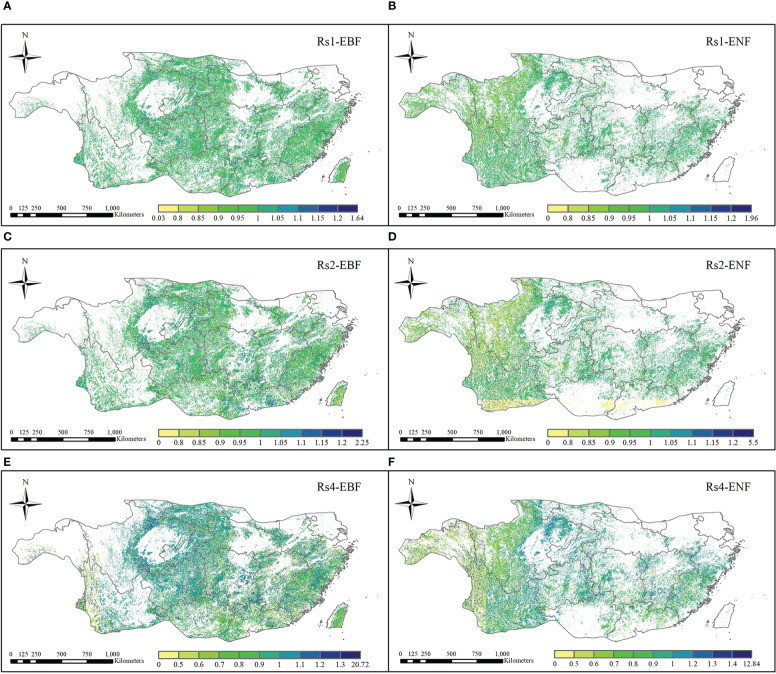
Spatial distribution of resilience of EBF and ENF to 2011 extreme drought after the first **(A, B)**, second **(C, D)**, and fourth **(E, F)** year.

**Figure 9** shows the spatial distribution of resilience of the EBF and ENF at 1, 2, and 4 years after the 2011 drought. As for the resilience of the two forest types one year after the drought, the median resilience of the EBF and ENF were 1.00 and 0.947, respectively (**Figure 7B**), which means that the resilience levels of the two forest types in the first year after drought were similar, with the resilience of the EBF being slightly stronger than that of the ENF. The spatial distribution of resilience of the EBF and ENF showed certain differences (**Figures 9A, B**). Overall, the spatial distribution of EBF resilience was relatively average, whereas the resilience of ENF was weak in the northwestern part of the subtropical region and strong in the central to eastern part.

In the second year after the 2011 drought (2013), the median resilience of the two forest types was 1.00 and 0.87, respectively (**Figure 7C**). The resilience level of the EBF was similar to that one year later, while the resilience level of the ENF slightly decreased. The spatial distribution of EBF resilience in the two years after the drought was similar to that in the year after the drought, which was relatively average overall (**Figures 9C, D**). However, the resilience level of the ENF in the southern subtropical region showed a clearly decreasing trend in the two years after the drought, which was clearly different from that in the northern region, which may be related to the uncertainty of carbon storage simulation.

In the fourth year (2015) after the 2011 drought, the resilience levels of the two forests were generally similar (**Figure 7D**), with median resilience values of 1.26 and 1.33, respectively, indicating that the resilience of both forests had increased. The spatial distribution of resilience at the fourth years after the drought is shown in **Figures 9 E, F**, the resilience levels of both forests in the central subtropical region were significantly higher than those in the western and southeastern subtropical regions, and higher levels of resilience areas were mainly distributed in Yunnan, Sichuan, Chongqing, Guizhou and other regions of the junction, which also represent areas that suffered more severe drought mentioned in Section 3.1. From the perspective of spatial distribution, the resilience of the two types of forests increased in the central subtropical region but decreased in the western subtropical region decreased. The inconsistency of resilience in different regions may be caused by the spatial heterogeneity of drought levels and the different spatial distributions of forests. Thus, the water-heat balance conditions of different pixels and the relationship between forest abundance data and the resilience level must be further compared. The resilience values indicate that both forest types will return to normal conditions within four years.

### 3.4 Effects of continuous drought on forest resilience

As mentioned above, changes in drought frequency affect forest resistance and resilience; therefore, this section examines the effects of continuous drought on forest resistance and resilience.

**Figure 10:**
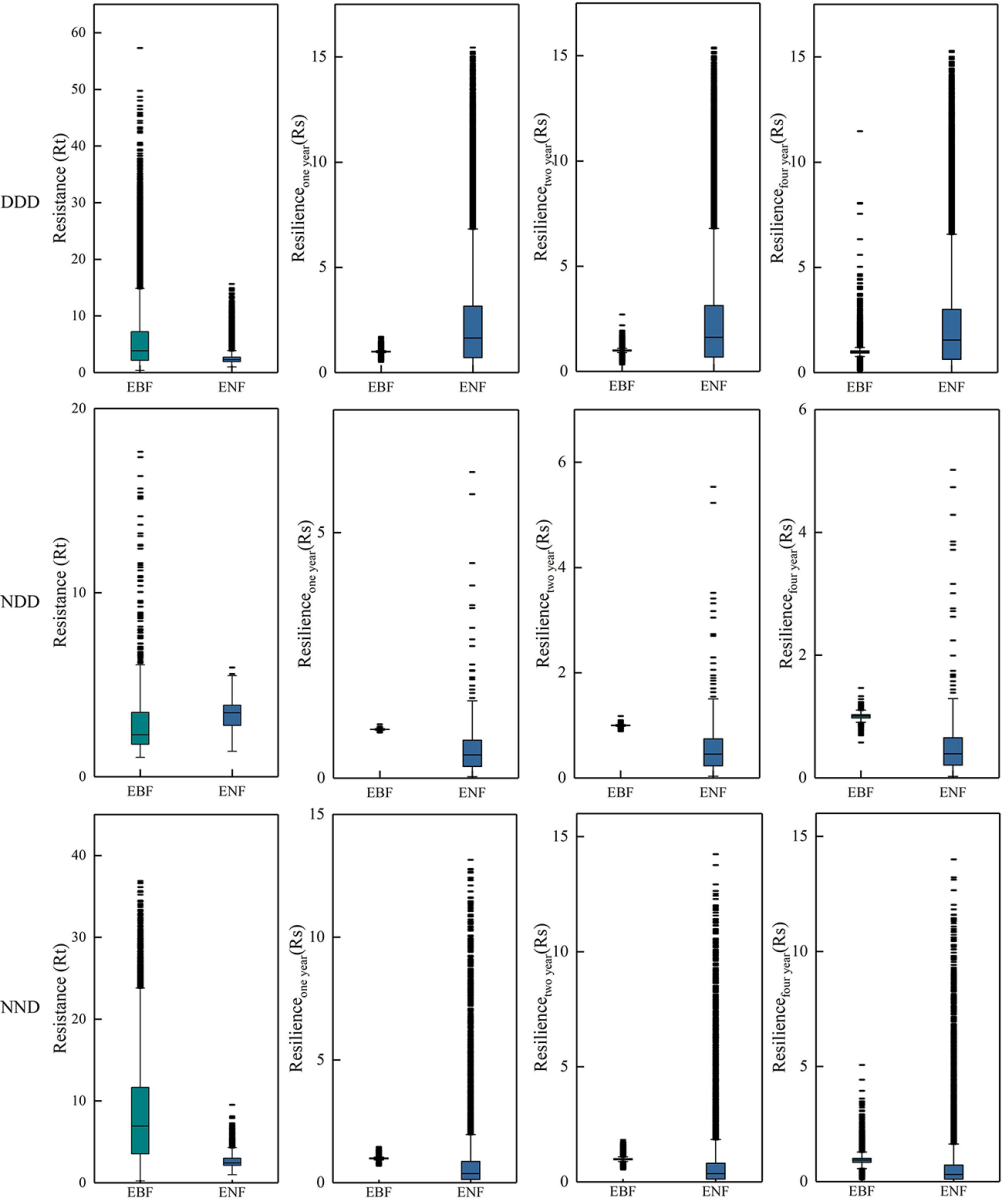
Statistical of Rt and Rs of two typical subtropical forests during and after the 2011 extreme drought. Each row represents a different combination of droughts. “N” represents normal, “D” represents drought, and “NDD” represents pixels that were normal in 2009 but drought in 2010 and 2011, i.e., pixels that suffered drought for two consecutive years. The first column shows the Rt in 2011, and columns two to four show the resilience of the first, second, and fourth years after the drought.

In this study, the year of extreme drought was 2011. Pixels that were or were not affected by drought in 2009, 2010, and 2011 were divided into two categories, and pixels that presented the two different situations in the three years were combined. The forest pixels that experienced three consecutive drought years (2009-2011), two consecutive drought years (2010-2011), and one drought year (2011) were screened and divided into three climatic combinations. **Figure 10** shows the resistance and resilience of the two forests under the three drought combinations.

**Figure 10** shows that the EBF generally has higher resistance under three different continuous drought conditions, that is, subtropical EBF in China can quickly adapt to drought. In terms of resilience, under the condition of a different combination of drought, two kinds of resilience showed similar levels of forest, and in the fourth year after the drought, two kinds of resilience of forest showed very similar levels, suggesting that the two kinds of forest will recover to normal levels after four years of drought. These results support the idea that forests need at least one year to recover from interannual droughts (Schwalm et al., 2017).

## 4 Discussion

### 4.1 Uncertainty analysis of SPEI calculation

The Thornthwaite (TW) (Thornthwaite, 1948) and P-M (Monteith, 1965) formulas were commonly used to calculate the PET in the SPEI estimation. The SPEI calculated based on TW formula was easily to implement, while it may indicate excessive dry conditions due to the influence of temperature, under the significantly increased temperatures in recent years (Zhang et al., 2017; Nouri and Homaee, 2020; Guo et al., 2022). Compared to the TW formula, which only considers temperature, the P-M formula considered variety of meteorological factors, which are more complex, and is generally more consistent with actual evapotranspiration (Jensen et al., 1990), specially it enable to describe the regions influenced by aerodynamic factors (Liu and Jiang, 2015). Consider, these two methods were widely used in the drought detection researches, further validation is required to determine which algorithm is better characterized in subtropical China.

The SPEI results were limited by meteorological stations, whereas some areas had few meteorological stations. Therefore, the accuracy of the SPEI in characterizing drought conditions in the study area needs to be confirmed by further comparative analysis with more observational data. The results of the temporal analysis indicate that the SPEI_12_ shows a significant downward trend from the 1950s to the 2010s, the interannual drought in the subtropical region of China shows an increasing trend from the 1950s to the 2010s, which is consistent with previous studies (Liu and Jiang, 2015). Spatial analysis showed a clear trend of drought in the subtropical southwest, especially in the Sichuan Basin, which is consistent with previous research findings (Li et al., 2012). A comparison of historical drought events showed that in 1982, Ningbo, Jinhua, Wenzhou, Jiujiang, and Nanchang (Zhang and Liu, 1993; Zhang et al., 2003); in 1986, Nanchang, Shangrao, Ji ‘an, and Ganzhou (Zhang and Liu, 1993; Zhang et al., 2003); in 1991, south Guangdong and Guangxi (Wang and Chen, 2012); in 1994, Suzhou and Shanghai (Zhang et al., 2003); and in 2000, moderate to severe drought in Yangzhou, Nanjing, Hefei and Anqing (Zhang et al., 2003). The simulation results of SPEI in this study were consistent with typical historical drought events (**Table 4**).

**Table 4:** Latitude and longitude of cities where historical drought events occurred.

Time	City	Latitude and longitude	Literature
1982	Ningbo	28°51’-30°33’N,120°55’-122°16’E	Zhang et al. (2003)
1982	Jinhua	28°32’-29°41’N,119°14’-120°46’30”E	Zhang et al. (2003)
1982	Wenzhou	27°03’-28°36’N, 119°37’-121°18’E	Zhang and Liu (1993)
1982	Jiujiang	28°47’-30°06’N,113°57’-116°53’E	Zhang and Liu (1993)
1982	Nanchang	28°10’-29°11’N,115°27’-116°35’E	Zhang and Liu (1993)
1986	Nanchang	28°10’-29°11’N,115°27’-116°36’E	Zhang and Liu (1993)
1986	Shangrao	27°48´-29°42´N,116°13´-118°29´E	Zhang and Liu (1993)
1986	Ji’an	25°58′-27°57′N, 113°46’-115°56’E	Zhang et al. (2003)
1986	Ganzhou	24°29′-27°09′N,113°54′-116°38′E	Zhang et al. (2003)
1994	Suzhou	30°47′-32°02′N,119°55′-121°20′E	Zhang et al. (2003)
1994	Shanghai	30°40′-31°53′N,120°52′-122°12′E	Zhang et al. (2003)
2000	Yangzhou	32°15′-33°25′N,119°01′-119°54′E	Zhang et al. (2003)
2000	Nanjing	31°14′-32°37′N, 118°22′-119°14′E	Zhang et al. (2003)
2000	Hefei	30°56′-32°33′N,116°40′-117°58′E	Zhang et al. (2003)
2000	Anqing	29°47′-31°16′N,115°45′-117°44′E	Zhang et al. (2003)

### 4.2 Simulation uncertainty of carbon storage in subtropical forests

The uncertainty of the Biome-BGC model may be divided into the uncertainty of the model structure, input variables, and uncertainty of model parameters (Li and Sun, 2018). First, the uncertainty of the model structure may be due to inadequate simulation of carbon, nitrogen, and water cycles in the ecosystem, which may lead to the difference between the simulation and observations (Churkina et al., 2003; Hidy et al., 2012; Smith and Dukes, 2013). Impacts like human activities (Du et al., 2021) (e.g., management practices and forest wildfires) on the carbon and nitrogen water cycle were not considered in this simulation, which may create uncertainty. Second, the uncertainty of the input variables may be due to errors or inadequacies in the collection and statistics of input data (Jung et al., 2007; Eastaugh et al., 2011). Although the forest abundance data used in this simulation are from fine-scale classification products with a resolution of 30 m, resampling to 1 km does not avoid pixel mixing and thus errors (Su et al., 2022). Third, the uncertainty of the model parameters may be due to their different effects of the model parameters on the output results under different conditions (Tatarinov and Cienciala, 2006; Kang, 2016; Raj et al., 2018). For some difficult-to-obtain parameters, this study was obtained by reviewing the literature and directly using the model defaults, which may cause uncertainty in the results.

**Figure 11:**
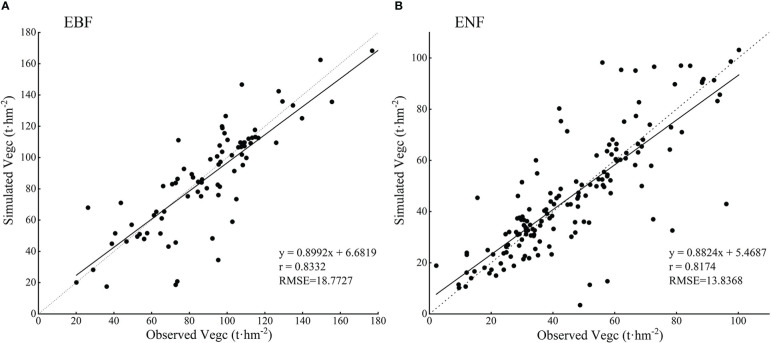
The comparison between simulated and observed vegetation carbon storage of two typical subtropical forests in Zhejiang Province (**A**. EBF and **B**. ENF).

However, by comparing the simulated values with the observed values in the Zhejiang forest inventory sample plots, and using evaluation indicators such as correlation coefficient and root mean square error to analyze the simulation accuracy, we found that the simulated carbon storage values of both forest types in this study were correlated with the observed values, as shown in **Figure 11**, and the mean values of carbon storage of both forest types were within the range of the values reported in previous studies (**Table 5**).

**Table 5:** Comparison between the mean values of carbon storage of two forest types simulated in this study and previous studies.

The time range	EBF(t·hm^-2^)	ENF(t·hm^-2^)	Literature
1985-2019	130.58	78.49	This study
1994	/	40	Gu et al. (2010)
1996	66.1	/	Minghong et al. (1996)
1999	26.3	/	Wang (1999)
2000	100.73	/	Zhou et al. (2000)
2000	/	80.798	Yun-Ting and Jiang-Ming (2002)
1977-2008	40.7	33.9	Yang et al. (2022)
1979-2012	/	21.37	Li et al. (2021)
2000-2014	74.2	62	Zhang et al. (2019b)
2004-2014	70.91	/	Peng et al. (2016)
2007	89.2	/	Zhang et al. (2007)
2008	53.62	/	Wang et al. (2021)
2009	/	12.72	Jin et al. (2019)
2009-2018	43.7	37.5	Yang et al. (2022)
2010	129.34	/	Zeng et al. (2020)
2010.5-6	134.9	/	Zeng et al. (2013)
2011	38.92	/	Hu et al. (2011)
2011	126	/	Zhang et al. (2020)
2012	97.49	/	Sun and Guan (2014)
2012-2013	97.30	/	Zhang et al. (2019a)
2010-2050	94.71	91.33	He et al. (2017)

### 4.3 Differences in ecosystem stability between EBF and ENF

This study shows the spatial differences between drought resistance and resilience of the EBF and ENF in the Chinese subtropics (**Figures 8**, **9**). As shown in the figure, the resistance value of the EBF to drought was significantly higher than that of the ENF, indicating that EBF has a stronger drought resistance ability in the subtropical region of China, which is consistent with previous studies (Huang and Xia, 2019; Shao et al., 2022). The reason may belong to the higher photosynthesis efficiency of EBF than that of ENF during drought (Wu and Wang, 2022). Previous studies found that EBF could accelerate the loss of old leaves and maintain the growth of young leaves to maintain the light use efficiency, and increase the carbon sequestration capability (Wu et al., 2016; Wei et al., 2017). Although there is significant difference in the resistance of the two forest types, no significant difference in resilience between the two forests 1-4 years after the drought. Previous studies found ecosystem resilience at a large scale can be expressed by their respective WUE levels (Ponce-Campos et al., 2013; Stan et al., 2021). Therefore, due to the similar change trend of WUE in the case of changes in hydroclimatic conditions of EBF and ENF (Sharma and Goyal, 2018; Huang and Xia, 2019), resulting the similar resilience of EBF and ENF to drought in subtropical China. However, the varies in climate and geological conditions cased spatial difference of WUE in two forests, which may be the reason of spatial heterogeneity in resilience throughout subtropical China (Guo et al., 2019; Shao et al., 2022).

EBF and ENF are the predominant vegetation types in subtropical regions of China, especially EBF, which accounts for approximately 60% of global photosynthetic carbon uptake (Mitchard, 2018). Their stability to drought plays a key role in maintaining the stability of subtropical forest ecosystems. According to the analysis results, there were certain differences in the ecosystem stability of the two types of forests, indicating that the stability of different biomes is different (Pennington and Lavin, 2016; Gazol et al., 2018; Anderegg et al., 2020). Current forest management and protection strategies (e.g. Natural forest resources protection, Returning farmland to forest, etc.) have made great improvement on carbon sequestration capability (Kong et al., 2020; Tong et al., 2020), and the increasing forest stand quality further enhanced the LUE and WUE of forest ecosystems (Ponce-Campos et al., 2013; Guo et al., 2019; Huang and Xia, 2019; Shao et al., 2022), leading to strong resistance and resilience to drought. There is no doubt that these strategies should continue to be implemented and widely promoted to provide strong support for subtropical forests to respond and adapt to climate change. However, whether the stability of evergreen forests could remain at current stage under the continuous increasing severe drought events should be further investigated in the future (Easterling et al., 2000; Dai, 2013; Reichstein et al., 2013).

## 5 Conclusion and recommendation

In this study, the FAO-PM algorithm was used to calculate SPEI data, and the Biome-BGC model was used to simulate carbon storage data. The spatiotemporal distribution characteristics of drought in the subtropical regions of China from 1952 to 2019 were analyzed, and the resistance and resilience of two types of forests to drought in the subtropical regions of China were quantified. The following conclusions were drawn:

1. From 1952 to 2019, China’s subtropical forests experienced five extreme drought events, with approximately one every 13.6 years. Two large-scale extreme drought events occurred after 1985 in 2009 and 2011, with 2011 being the year with the most severe and widespread drought. In the EBF, 19.3% suffered from extreme drought and 26.6% suffered from severe drought. In the ENF, 31.9% suffered from extreme drought and 21.2% suffered from severe drought.
2. From 1985 to 2019, the average carbon storage of vegetation in EBF and ENF in the subtropical region of China was 130.58 t·hm^-2^ and 78.49 t·hm^-2^, respectively. From 2011 to 2015, the mean values of the overall change trend of carbon storage of the EBF and ENF in the subtropical region of China were -0.042 a^-1^ and -0.013 a^-1^, respectively. The carbon storage of vegetation in both forests showed a slight downward trend, and the spatial distribution of changes in carbon storage was complex.
3. There were significant differences in the resistance of the two forest types to extreme drought, with EBF being significantly more resistant to drought than ENF in subtropical China, and EBF and ENF were broadly similar in resilience levels after drought. Therefore, the EBF is better adapted to drought in the subtropical region of China, and its high stability is mainly due to its high resistance to drought. The results indicating that better management level or extend the EBF plantation to increase the proportion of EBF in subtropical forest may enhance the resistance and resilience of the region to severe drought.

## Data availability statement

The raw data supporting the conclusions of this article will be made available by the authors, without undue reservation.

## Author contributions

MY: Data curation, Formal analysis, Investigation, Methodology, Validation, Preparation of the first draft. FM: Conceptualization, Methodology, Data Curation, Formal Analysis, Funding Acquisition, Review and editing. HD: Funding Acquisition, Supervision. XL: Methodology, Formal analysis, Data curation. QC: Formal analysis, Investigation. CN:Formal analysis, Investigation. ZH: Formal analysis, Investigation. YX: Formal analysis, Investigation. YG:Formal analysis, Investigation. KG:Formal analysis, Investigation. JS:Formal analysis, Investigation. CX:Formal analysis, Investigation. All authors contributed to the article and approved the submitted version.
